# CircRNAs expression profile and potential roles of circRERE-PMN in pre-metastatic lungs

**DOI:** 10.3389/fimmu.2024.1455603

**Published:** 2024-08-26

**Authors:** Huifang Shi, Yan Wang, Lei Chen, Yuanyuan Li, Yan Qin, Jie Lv

**Affiliations:** ^1^ Clinical Laboratory, The Rizhao People’s Hospital Affiliated to Jining Medical University, Rizhao, Shandong, China; ^2^ CT Scan Room, The Rizhao People’s Hospital Affiliated to Jining Medical University, Rizhao, Shandong, China

**Keywords:** cancer, lung metastasis, circRNAs, pre-metastatic niche, inflammation

## Abstract

The successful pulmonary metastasis of malignant cancer cells depends on the survival of circulating tumor cells in a distant and hostile microenvironment. The formation of a pre-metastatic niche (PMN) creates a supportive environment for subsequent metastasis. Circular RNAs (circRNAs) are increasingly acknowledged as crucial elements in the mechanisms of metastasis due to their stable structures and functions, making them promising early metastasis detection markers. However, the specific expression patterns and roles of circRNAs in the lungs before metastasis remain largely unexplored. Our research aims to chart the circRNA expression profile and assess their impact on the lung PMN. We developed a lung PMN model and employed comprehensive RNA sequencing to analyze the differences in circRNA expression between normal and pre-metastatic lungs. We identified 38 significantly different circRNAs, primarily involved in metabolism, apoptosis, and inflammation pathways. We then focused on one specific circRNA, circ:chr4:150406196 – 150406664 (circRERE-PMN), which exhibited a significant change in expression and was prevalent in myeloid-derived suppressor cells (MDSCs), alveolar epithelial cells, and macrophages within the pre-metastatic lung environment. CircRERE-PMN was found to potentially regulate apoptosis and the expression of cytokines and chemokines through its interaction with the downstream target HUR in alveolar epithelial cells. Overall, our study highlights the crucial role of circRNAs in the formation of lung PMNs, supporting their potential as diagnostic or therapeutic targets for lung metastasis.

## Introduction

Pulmonary metastasis represents the primary occurrence of malignant cancer-related metastasis (1). Conventional treatments for metastatic tumors include surgical resection, radiotherapy, chemotherapy, adjuvant chemotherapy, and targeted therapy. Despite these treatments, mortality rates remain high due to therapeutic resistance and toxic side effects (2). Consequently, there is an urgent need for further research to elucidate the mechanisms underlying pulmonary metastasis and to identify new potential targets for clinical diagnosis and treatment. The formation of a pre-metastatic niche (PMN) is crucial for facilitating the arrest, survival, and proliferation of circulating tumor cells. The PMN is characterized by various changes, including inflammation, immunosuppression, organotropism, angiogenesis/vascular permeability, lymphangiogenesis, and reprogramming, all of which support tumor cell colonization and metastasis (3). However, the regulatory mechanisms of PMN remain inadequately understood.

Circular RNAs (circRNAs) are a type of noncoding RNA formed from mRNA back-splicing or exon skipping, resulting in covalently closed loops without 5’ caps or 3’ polyadenylated tails (4, 5). They have been reported to function as miRNA sponges or influence alternative RNA splicing, thereby regulating gene expression (6). Additionally, circRNAs can also enhance the transcription of their parental genes and act as protein scaffolds or antagonists by binding to RNA-binding proteins (RBPs) (7). These diverse regulatory roles of circRNAs suggest their potential involvement in various diseases, including cancer progression. Increasing evidence underscores the importance of circRNAs in cancer (8). For instance, circ_0072995, acting as a miR-147 sponge, reduces miR-147’s inhibitory effect on its target CDK6, thereby promoting epithelial ovarian cancer cell proliferation, migration, and progression (9). The N6-methyladenosine (m6A) modification of circNSUN2 stabilizes HMGA2 mRNA, enhancing colorectal carcinoma metastasis by forming a circNSUN2/IGF2BP2/HMGA2 RNA-protein complex (10). Notably, circRNAs play significant roles in the multifaceted functions of the tumor microenvironment (11). They regulate tumor immunity by modulating the antitumor immunity of immune cells, the cytotoxicity of NK cells (12), and the activity and proliferation of T cells (13, 14). Furthermore, circRNAs influence endothelial monolayer permeability and tumor angiogenesis (15, 16). Given their crucial role in the tumor microenvironment, it is plausible that circRNAs are essential for PMN construction and exert significant functions. For example, circIKBKB was verified to promote the establishment of bone PMN through modulating breast cancer cells (17); Elevated expression of circ-ZNF609 in HUVECs was linked to enhanced angiogenesis and vascular permeability, thereby facilitating PMN development in esophageal squamous cell carcinoma (18). However, the global expression profile of circRNAs and their regulatory roles in pre-metastatic lungs remain to be fully elucidated.

In this study, we analyzed circRNA expression profiles in pre-metastatic and normal lungs of mice using RNA sequencing. RT-qPCR was employed to validate the aberrant expression of the top 6 circRNAs. Gene ontology (GO) and Kyoto Encyclopedia of Genes and Genomes (KEGG) analyses were conducted to uncover potential biological processes involved in the formation of the pre-metastatic lung microenvironment. Additionally, we identified that circ:chr4:150406196–150406664 (circRERE-PMN) was essential for apoptosis and expression of cytokines and chemokines in alveolar epithelial cells.

## Materials and methods

### Mice and cell lines

C57BL/6J female mice (8-10 weeks old) were purchased from Animal Experimental Center of Shandong Province (Jinan, China). All mice were maintained under protocols approved by the Animal Care Committee of Jining Medical College in a specific pathogen-free environment, and all experiments correlated with the mice were carried out according to the approved guidelines (approval number: JNMC-2024-DW-069). B16F10 and MLE-12 cell lines were obtained from the American Type Culture Collection. The B16F10 cells were cultured in DMEM (GIBCO, NY, USA) supplemented with 10% FBS (HyClone, Logan, UT, USA), and DMEM/F-12 (GIBCO, NY, USA) with 10% FBS were for MLE-12 cells.

### Tumor model establishment and lung specimen collection

To establish a pre-metastatic lung tumor model, 1×10^6^ B16F10 cells were subcutaneously inoculated into the flanks of C57BL/6J mice, as outlined in previous research (19). Two weeks subsequent to the cells inoculation, lung tissues from both the treated and untreated mice were obtained after perfusing with pre-cooled PBS to remove the circulating peripheral blood cells. The lung tissues were then immediately frozen in liquid nitrogen.

### RNA extraction and high-throughput sequencing analysis

Total RNA from freshly frozen tissues was extracted with TRIzol reagent (Invitrogen) according to the manufacturer’s instructions. Then the RNA concentration and purity of each sample were assessed using NanoDrop 2000, and their integrity was verified via Agilent2100/LabChip GX Assay, with an OD260/OD280 ratio of 1.8 to 2.0 deemed suitable for further experiment. RNA-seq library was prepared and sequencing was carried out on an Illumina platform in PE150 mode. Clean data was obtained from raw data by filtering reads with low quality. Bioinformatic analysis was performed using BMKCloud (www.biocloud.net).

### Cell proliferation assay

The proliferation of MLE-12 cells was examined by CCK8 assay. In brief, MLE-12 cells transfected with siRNA or not were seeded into 96-well plates. Then, 10 μl CCK8 reagent was added to each well at indicated time, followed by incubation at 37°C for 1 hour. Subsequently, the absorbance values were measured at 450nm in a microplate absorbance reader (BIO-RAD iMark).

### RT-qPCR

The extracted total RNA was reverse transcribed into cDNA using the PrimeScript RT Reagent Kit from TaKaRa (China). RT-qPCR was performed using SYBR Green Master Mix (TaKaRa, China) on an ABI 7500. The difference in cycle threshold values (2^-ΔΔCT^) was calculated to evaluate the relative expression of genes, normalizing against β-actin. The primer sequences utilized are listed in **Table 1**.

**Table 1:** Primers used for RT-qPCR.

Genes	Primer sequence (5’-3’)
CXCL1	Forward: ATGGCTGGGATTCACCTCAA
CXCL1	Reverse: CAAGGGAGCTTCAGGGTCAA
CXCL2	Forward: GCCCAGACAGAAGTCATAGCC
CXCL2	Reverse: TCAGTTAGCCTTGCCTTTGTTC
S100A8	Forward: TGCCCTCTACAAGAATGACT
S100A8	Reverse: CTTGTGGCTGTCTTTGTGAG
S100A9	Forward: CCAACAAAGCACCTTCTCAG
S100A9	Reverse: TTGCCATCAGCATCATACAC
IL-1β	Forward: TGACGTTCCCATTAGACAACTG
IL-1β	Reverse: CCGTCTTTCATTACACAGGACA
Bv8	Forward: GCATGACAGGAGTCATCATTTT
Bv8	Reverse: AAATGGCAGGATATCAGGAAA
MMP9	Forward: CTTCTGGCGTGTGAGTTTCCA
MMP9	Reverse: ACTGCACGGTTGAAGCAAAGA
β-actin	Forward: AACAGTCCGCCTAGAAGCAC
β-actin	Reverse: CGTTGACATCCGTAAAGACC
circ4:126814256-126816474 (circAU040320-PMN)	Forward: TTCTCAGAGCACCTCAGCAC
circ4:126814256-126816474 (circAU040320-PMN)	Reverse: CTCGGATGGTTCAGGATGGATG
circ16:93849130-93853898 (circMORC3-PMN)	Forward: ACGTGATGTTTACCGACCTA
circ16:93849130-93853898 (circMORC3-PMN)	Reverse: TCTTGTACCCTTTCCTCCCT
circ19:38523915-38525452 (circPLCE1-PMN)	Forward: AACTCTGCCTTCTGGCTGTA
circ19:38523915-38525452 (circPLCE1-PMN)	Reverse: TCTGCGACTTGTGGCTTATT
circ6:52619977-52630349 (circHIBADH-PMN)	Forward: CAATGCTGCCCTCCAGTATGA
circ6:52619977-52630349 (circHIBADH-PMN)	Reverse: TTCCCAGTCCAATGAATCCAAC
circ4:150406196-150406664 (circRERE-PMN)	Forward: GACAACAACAGCGCCACCAC
circ4:150406196-150406664 (circRERE-PMN)	Reverse: CTCCGTGAAAGGTAGACAGTGAGC
circ9:76996893-77027004 (circTINAG-PMN)	Forward: AAAATGGACCAGTTCAAGCC
circ9:76996893-77027004 (circTINAG-PMN)	Reverse: AGATCAGCTCTGGGAGGAAA

### Single cell preparation and flow cytometry

After perfusion with PBS to clear the blood vessels, the harvested lungs were minced and digested with collagenase I (2mg/ml) at 37°C for 1 hour, and then the digested tissue was strained through a 149 μm mesh to yield a single-cell suspension. Then, the cells were stained with fluorochrome-conjugated antibodies after pre-treated with the blocking solution (PBS+10% FBS). Flow cytometry analysis was performed in a FACSCantoII (BD Biosciences), and the results were processed with Flow Jo software (version 7.6.1).

### SiRNA transfection

SiRNAs targeting the circRERE-PMN sequence and control siRNA were obtained from GenePharma, detailed in **Table 2**. Lipofectamine 3000 (Invitrogen) was employed to transfect the siRNAs into the cells. 5×10^5^ MLE-12 cells were seeded in 6-well plates and proceeded with transfection the following day. The transfected cells were collected at least 24h after transfection for subsequent analysis.

**Table 2:** SiRNA sequences.

Target genes	Sequence
circRERE-PMN^#^1	Sense: CUACAGACCGGGAGAAUAUTT
circRERE-PMN^#^1	Antisence: AUAUUCUCCCGGUCUGUAGTT
circRERE-PMN^#^2	Sense: ACAGACCGGGAGAAUAUUUTT
circRERE-PMN^#^2	Antisence: AAAUAUUCUCCCGGUCUGUTT

### Fluorescence *in situ* hybridization and fluorescence immunohistochemistry staining

The cellular disposition of circRERE-PMN in pre-metastatic lungs were conducted via fluorescence *in situ* hybridization (FISH). The circRERE-PMN probe 5’-TCAAATAT+TCTCCCGGTCTGTAGAC-3’, tagged with Cy3, was incubated at 37°C overnight, succeeded by a session with cell-specific markers, and finally stained with FITC-linked secondary antibodies for one hour at room temperature. DAPI staining was used to visualize the nuclei of cells, and a fluorescence microscope was employed to detect the emitted signals (3DHISTECH).

### Statistical analysis

In terms of data analysis, the results were evaluated using the Student’s t-test and expressed as mean values with standard error of the mean. A P-value of less than 0.05 was the threshold for statistical significance.

## Results

### The construction of an inflammatory pre-metastatic microenvironment before lung metastasis of melanoma cells

**Figure 1:**
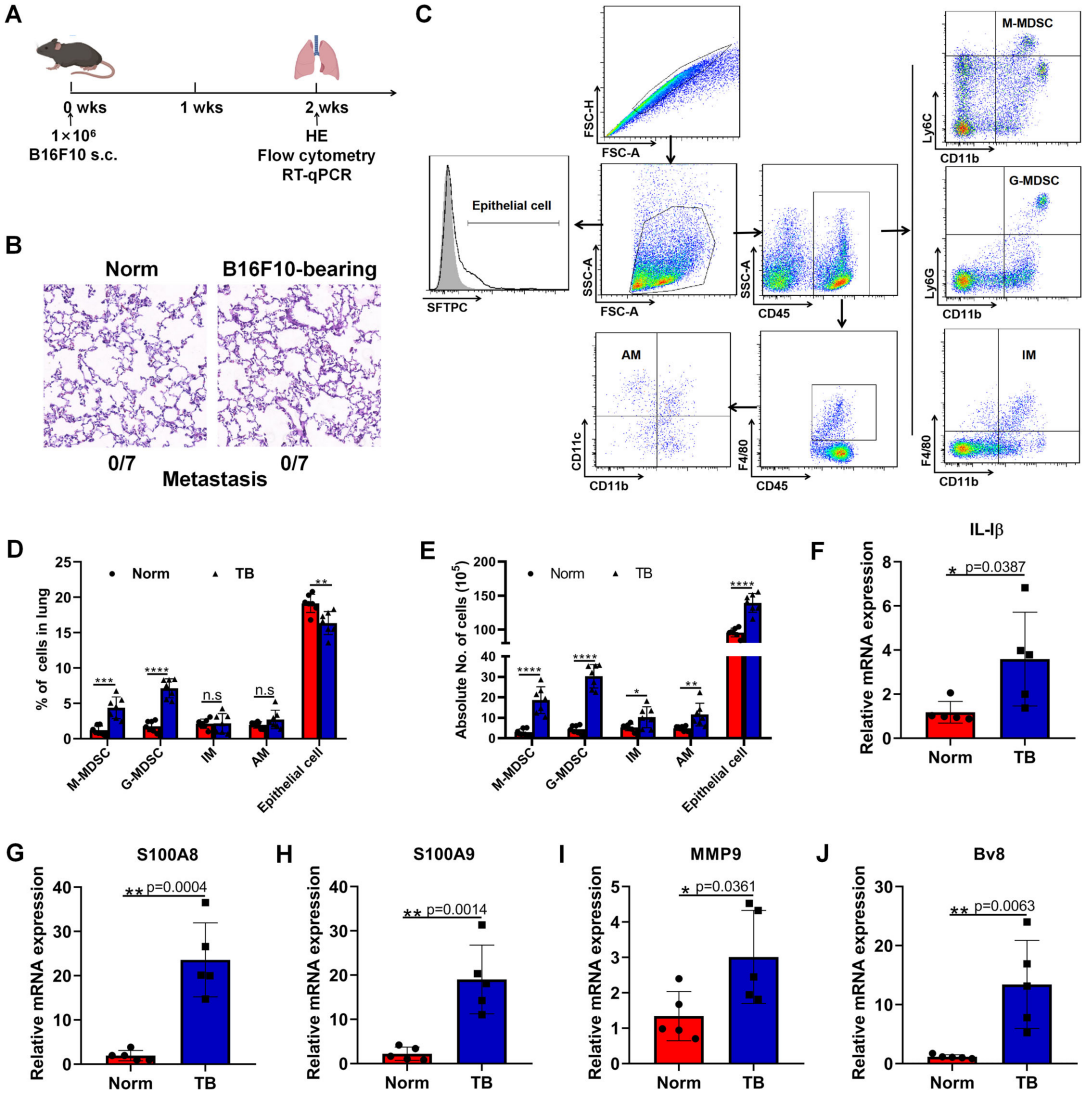
Subcutaneous inoculation of B16F10 cells induces a pre-metastatic niche in the lung. **(A)** Female 8-10 weeks-old C57BL/6J mice were subcutaneously inoculated with 1×10^6^ B16F10 melanoma cells. Two weeks after inoculation, HE, flow cytometry and RT-qPCR were conducted. N=7, per group. **(B)** Detection of pulmonary metastasis by H&E staining 2 weeks post subcutaneous injection of 1×10^6^ B16F10 melanoma cells. Scale bar, 100 μm. **(C)** Phenotypic analysis of M-MDSCs, G-MDSCs, IMs, AMs and epithelial cells in the lungs. M-MDSCs: CD45^+^CD11b^+^Ly6C^+^, G-MDSCs: CD45^+^CD11b^+^Ly6G^+^, IMs: CD45^+^CD11b^+^F4/80^+^, AMs: CD45^+^F4/80^+^CD11b^-^CD11c^+^, epithelial cells: SFTPC^+^. **(D)** The proportions of M-MDSCs, G-MDSCs, IMs, AMs and epithelial cells were compared between normal and pre-metastatic lungs. N=7, per group. **(E)** The absolute counts of M-MDSCs, G-MDSCs, IMs, AMs and epithelial cells were compared between normal and pre-metastatic lungs. N=7, per group. **(F–J)** RT-qPCR analysis of IL-1β **(F)**, S100A8 **(G)**, S100A9 **(H)**, MMP9 **(I)**, and Bv8 **(J)** expression in the normal and pre-metastatic lungs. N=5, per group. *p < 0.05, **p < 0.01, ***p < 0.001, ****p < 0.0001, n.s, no significance.

To define the phase of pre-metastatic microenvironment in our experimental model, 1×10^6^ B16F10 melanoma cells, which were prone to metastasize to lung, were subcutaneously inoculated into the flanks of C57BL/6 mice (**Figure 1A**). Two weeks post-inoculation, H&E staining revealed no micro-metastases (0/7) in the lungs (**Figure 1B**). The pre-metastatic microenvironment was defined by an increased accumulation of myeloid cells and inflammatory cytokines. Consequently, hallmark cells and cytokines were analyzed two weeks post-implantation using flow cytometry and RT-qPCR. We observed a significant rise in the proportions of M-MDSCs (CD45^+^CD11b^+^Ly6C^+^) and G-MDSCs (CD45^+^CD11b^+^Ly6G^+^) in the lungs of tumor-bearing mice compared to normal mice, along with increased absolute numbers of M-MDSCs, G-MDSCs, interstitial macrophages (IMs) (CD45^+^F4/80^+^CD11b^+^), and alveolar macrophages (AMs) (CD45^+^F4/80^+^CD11b^-^CD11c^+^) (**Figures 1C–E**). Additionally, although the relative proportion of epithelial cells (SFTPC^+^) decreased, their absolute number increased in the lungs of tumor-bearing mice (**Figures 1C–E**). Genes associated with the formation of the PMN, such as IL-1β, S100A8, S100A9, MMP9, and Bv8, were significantly upregulated (**Figures 1F–J**). Together, these data indicated the establishment of an inflammatory pre-metastatic lung environment following B16F10 cell implantation.

### Identification of circRNA profiles in pre-metastatic lungs

**Figure 2:**
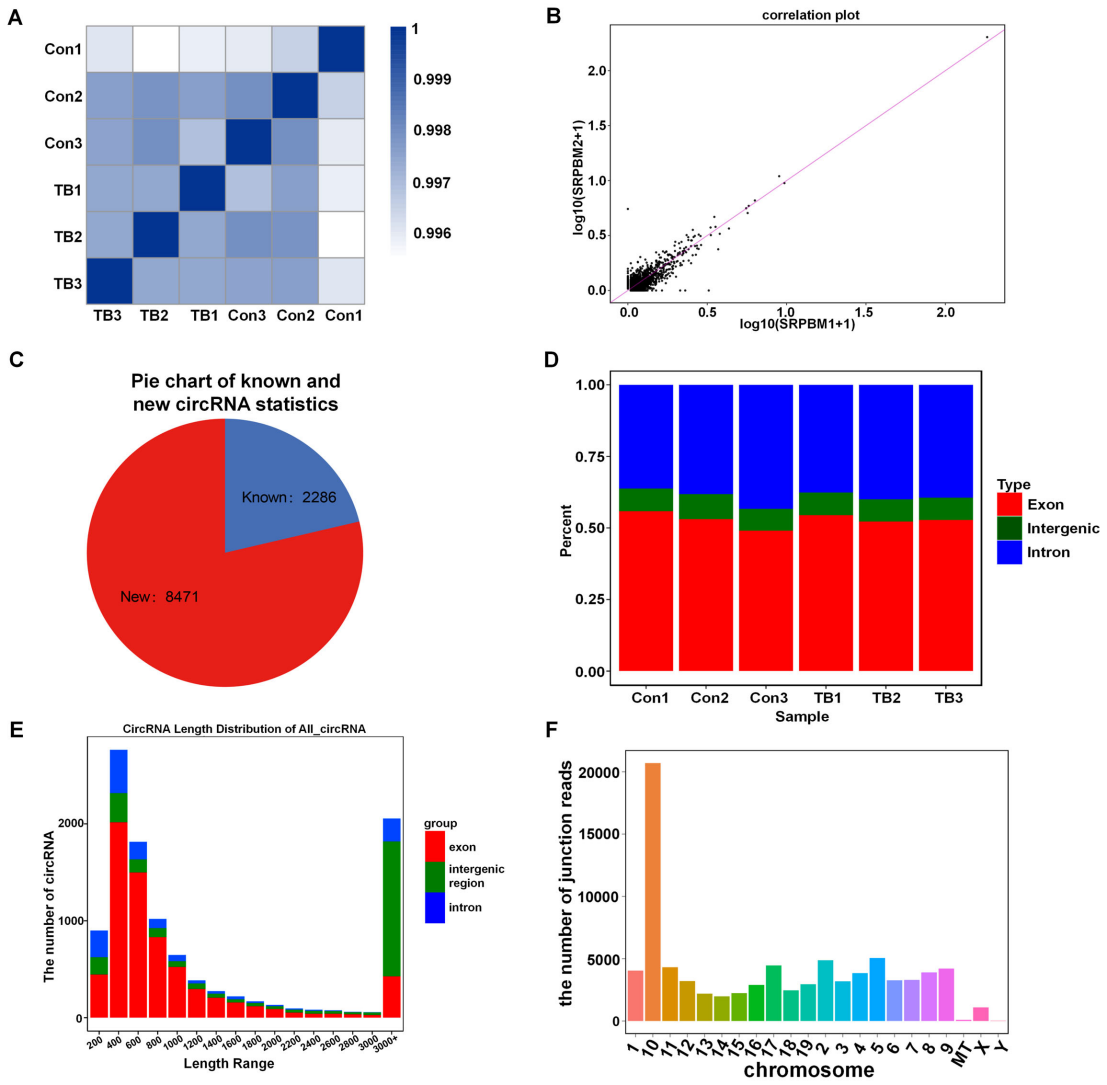
The analysis of circRNAs expression in normal versus pre-metastatic lung tissues. **(A)** A heat map displays the correlation between all six samples studied. **(B)** A correlation plot analysis was performed for the samples. **(C)** A pie chart categorizes known and new circRNAs in the normal and pre-metastatic lungs. **(D)** The genomic distribution percentage of circRNAs in each sample is shown. **(E)** The length distribution of all circRNAs is depicted. **(F)** The junction read counts of circRNAs in the chromosomes are enumerated.

RNA sequencing was used to analyze circRNA expression patterns in normal and pre-metastatic lung tissues. Initially, the quality of the samples was evaluated using the Q30 score (a correct value is over 99.9%), and all samples exhibited a Q30 base percentage above 94.74% (**Table 3**). The biological correlation of the samples was then assessed with the Pearson correlation coefficient, showing strong correlations with r^2^ values exceeding 0.996 (**Figure 2A**). Additionally, a correlation diagram confirmed the experiment’s repeatability and sequencing reliability, with minimal deviation from the diagonal line (**Figure 2B**). The sequencing identified 10,757 circRNAs, of which 2,286 were previously known and 8,471 were newly discovered (**Figure 2C**). Approximately half of the sequencing reads covered the genomic exons, and the circRNA coverage varied in genomic length; exonic and intronic circRNAs mostly ranged from 400 to 600 nucleotides, while those from intergenic regions generally exceeded 3000 nucleotides (**Figures 2D, E**). Furthermore, circRNAs showed a locational preference on chromosome 10 (**Figure 2F**).

**Table 3:** Evaluation of sample sequencing data.

Samples	BMK-ID	Read Number	Base Number	GC Content	≥Q30
Control 1	Con 1	49,006,479	14,638,313,084	49.14%	94.74%
Control 2	Con 2	50,780,671	15,130,317,372	49.19%	94.98%
Control 3	Con 3	52,379,989	15,621,645,818	47.79%	95.36%
Tumor-bearing 1	TB 1	52,440,949	15,635,155,250	47.29%	95.27%
Tumor-bearing 2	TB 2	54,592,455	16,283,131,48	48.03%	94.92%
Tumor-bearing 3	TB 3	54,730,988	16,324,663,140	48.34%	94.95%

### The differential expression of circRNAs and functional annotation and enrichment analysis prediction in pre-metastatic lungs

**Figure 3:**
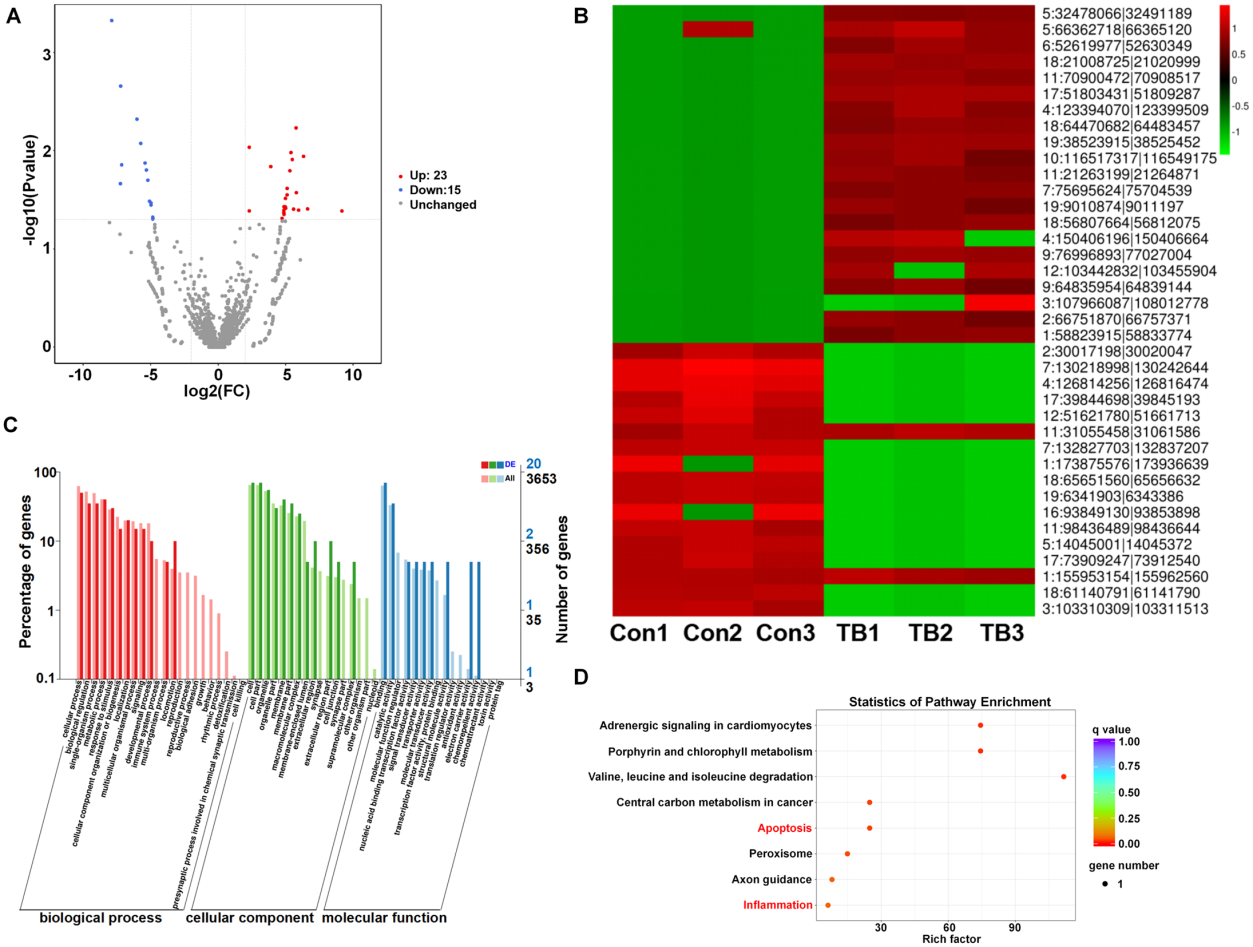
The differential expression and KEGG pathway analysis of circRNAs between normal and pre-metastatic lungs. **(A)** Volcano plots illustrate the differentially expressed circRNAs. **(B)** A hierarchical clustering heatmap provides an analysis of circRNA patterns. **(C)** Gene ontology enrichment analyses for the differentially expressed circRNAs. **(D)** Differentially expressed circRNAs were subjected to KEGG pathway analysis.

To analyze the altered expression of circRNAs in pre-metastatic lung tissues, we applied criteria of a fold change of 1.5 or greater and a p-value below 0.05. This analysis identified 38 circRNAs with altered regulation—23 upregulated and 15 downregulated—compared to normal lung samples, as shown in the volcano plot (**Figure 3A**). Hierarchical clustering further emphasized the circRNAs that were distinctly different between the two sample groups (**Figure 3B**). Given that circRNAs have the potential to regulate the expression of their source genes by modifying the epigenetic landscape of gene promoter regions or facilitating pre-mRNA circularization (20), the prediction of functions for the host genes of differentially expressed circRNAs was conducted. GO analysis for the differential circRNAs revealed that the most enriched items for biological processes were cellular process and metabolic process; binding and catalytic activity were the most enriched terms for molecular function (**Figure 3C**). Additionally, KEGG pathway analysis revealed their participation in essential biological pathways such as metabolism, apoptosis, and inflammation, indicating their potential role in PMN (**Figure 3D**).

### RT-qPCR verification of the differential expression of circRNAs

**Figure 4:**
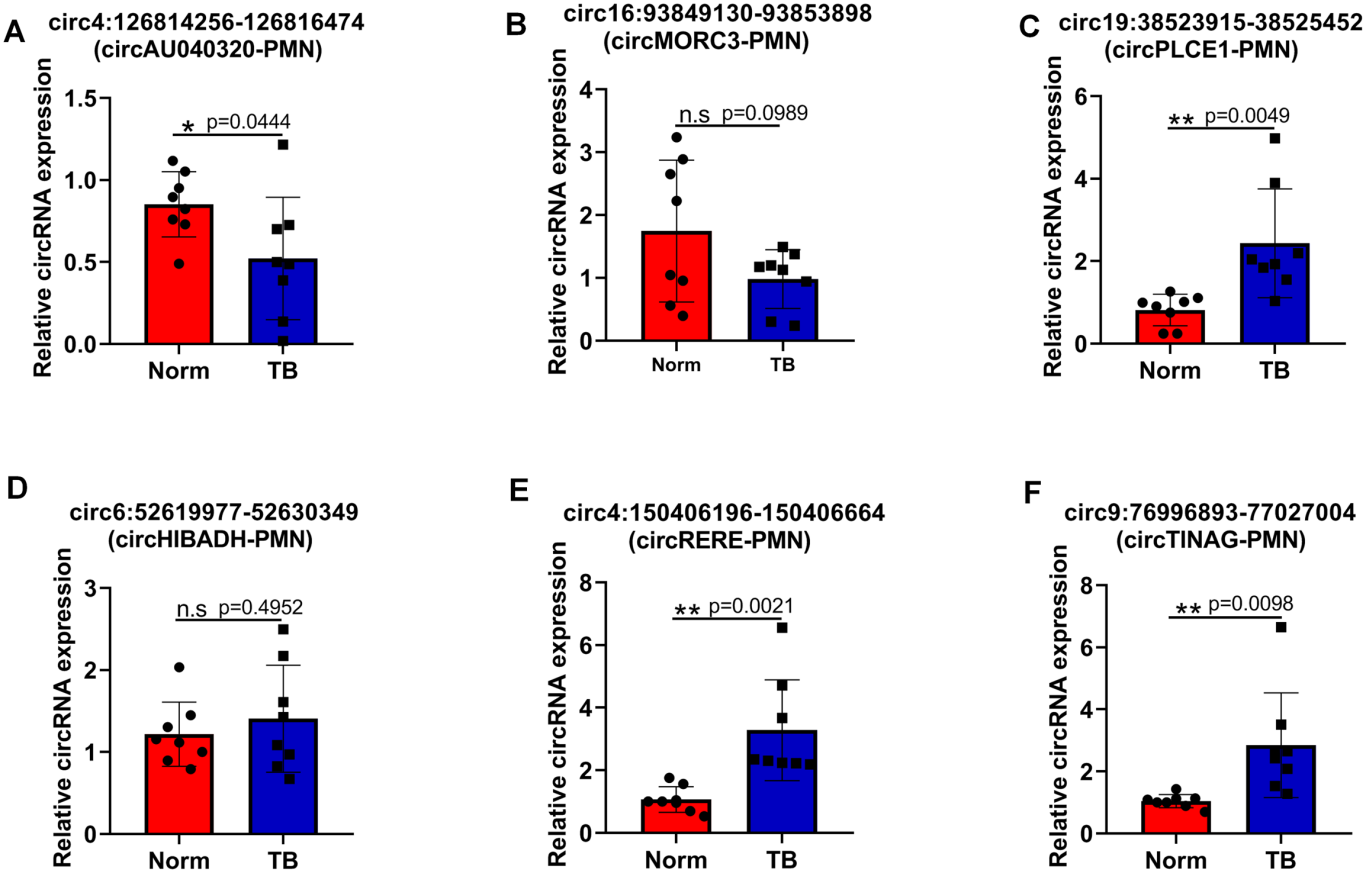
Verification of the differential expression of six selected circRNAs via RT-qPCR. **(A–F)** The relative expression levels of circ4:126814256-126816474 (circAU040320-PMN) **(A)**, circ16:93849130-93853898 (circMORC3-PMN) **(B)**, circ19:38523915-38525452 (circPLCE1-PMN) **(C)**, circ6:52619977-52630349 (circHIBADH-PMN) **(D)**, circ4:150406196-150406664 (circRERE-PMN) **(E)**, and circ9:76996893-77027004 (circTINAG-PMN) **(F)**. N=8, per group. *p < 0.05, **p < 0.01, n.s, no significance.

To validate RNA sequencing results, the top 6 candidate circRNAs were selected for RT-qPCR analysis. RT-qPCR confirmed the upregulation of circ:chr19:38523915 – 38525452 (circPLCE1-PMN), circRERE-PMN, and circ:chr9:76996893 – 77027004 (circTINAG-PMN), and the downregulation of circ:chr4:126814256 – 126816474 (circAU040320-PMN) in pre-metastatic lung samples. However, the expression differences of circ:chr16:93849130 – 93853898 (circMORC3-PMN) and circ:chr6:52619977 – 52630349 (circHIBADH-PMN) were not statistically significant between the two groups (**Figures 4A–F**). Notably, circRERE-PMN showed the most significant difference, highlighting its potential role in influencing the pre-metastatic microenvironment. Therefore, subsequent functional assays mainly focused on circRERE-PMN.

### CircRERE-PMN is widely distributed in MDSCs, alveolar epithelial cells and macrophages of pre-metastatic lungs

**Figure 5:**
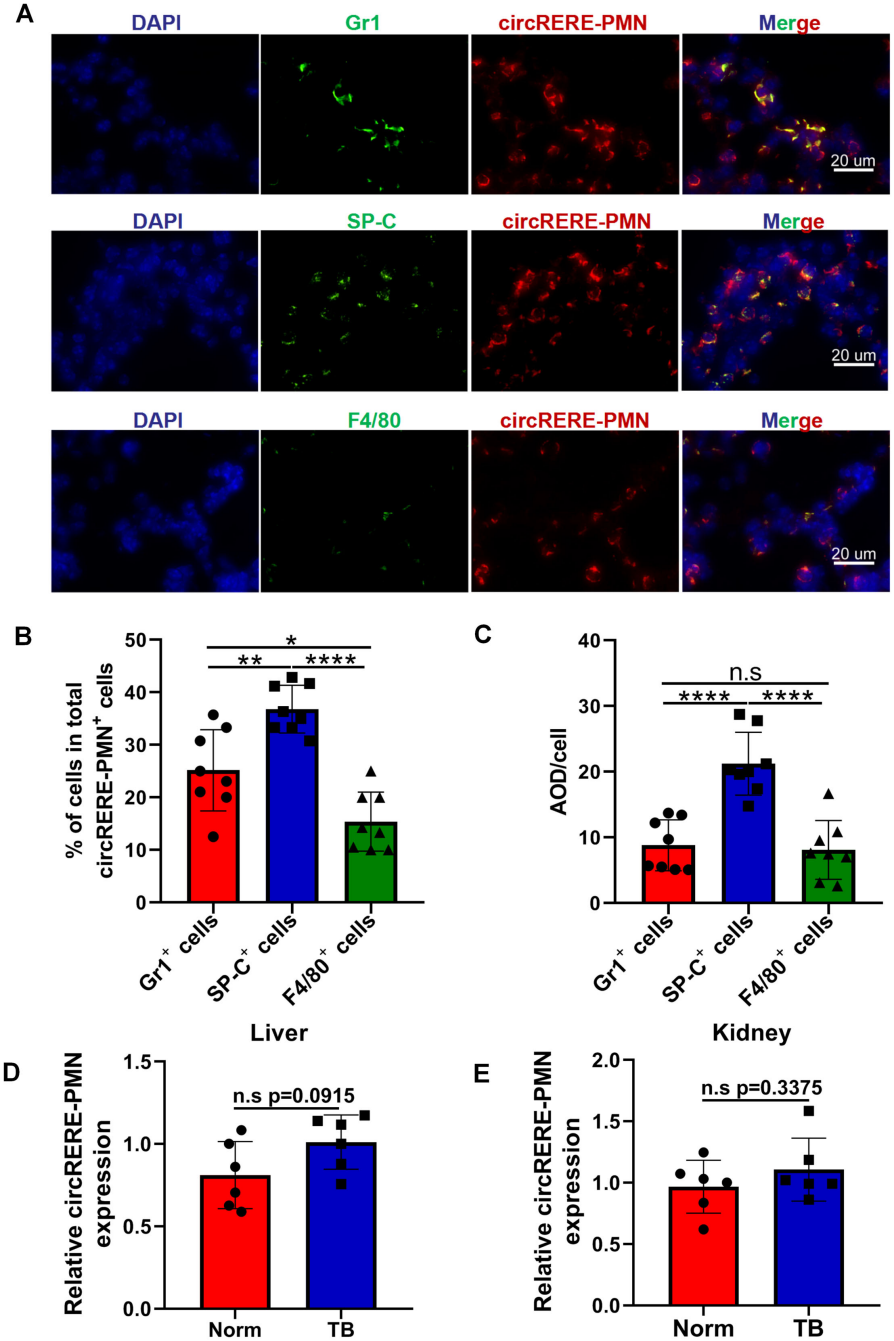
The distribution and relative expression levels of circRERE-PMN within MDSCs, alveolar epithelial cells and macrophages. **(A)** FISH and immunofluorescence techniques were used to co-localize circRERE-PMN (red) with specific cellular markers (green) in pre-metastatic lung tissue. Scale bar, 20 μm. **(B)** The proportion of circRERE-PMN positive cells was quantitatively assessed. **(C)** Quantitative analysis of the average optical density (AOD) of cells. N=8, per group. **(D, E)** RT-qPCR analysis the relative expression level of circRERE-PMN in liver **(D)** and kidney **(E)** between normal mice and tumor-bearing mice. N=6, per group. *p < 0.05, **p < 0.01, ****p < 0.0001, n.s, no significance.

Next, we aim to identify the specific cells in which circRERE-PMN is active. As depicted in **Figure 1D**, there was a significant increase in the numbers of MDSCs (including M-MDSCs and G-MDSCs), macrophages (including IM and AM), and alveolar epithelial cells in pre-metastatic lungs. Consequently, the expression of circRERE-PMN was measured in these three types of cells in pre-metastatic lung tissue. Immunofluorescence analysis revealed high expression of circRERE-PMN in MDSCs, macrophages, and alveolar epithelial cells, predominantly in the cytoplasm (**Figure 5A**). Quantitative analysis demonstrated a significantly higher proportion of alveolar epithelial cells among the total circRERE-PMN^+^ cells compared to MDSCs (**Figure 5B**). Furthermore, the proportion of macrophages within these cells was considerably lower than that of MDSCs in the total circRERE-PMN^+^ population (**Figure 5B**). Statistical analysis also showed that alveolar epithelial cells exhibited a markedly higher average optical density (AOD) compared to MDSCs and macrophages (**Figure 5C**). Collectively, these findings indicate a predominant presence of circRERE-PMN^+^ cells in MDSCs, alveolar epithelial cells, and macrophages in pre-metastatic lungs, with alveolar epithelial cells making the most significant contribution. Moreover, the relative expression levels of circRERE-PMN in liver and kidney were also examined, and no significant discrepancies were detected between two groups, further addressing its importance for lung metastasis (**Figures 5D, E**).

### CircRERE-PMN inhibits the apoptosis of alveolar epithelial cells

**Figure 6:**
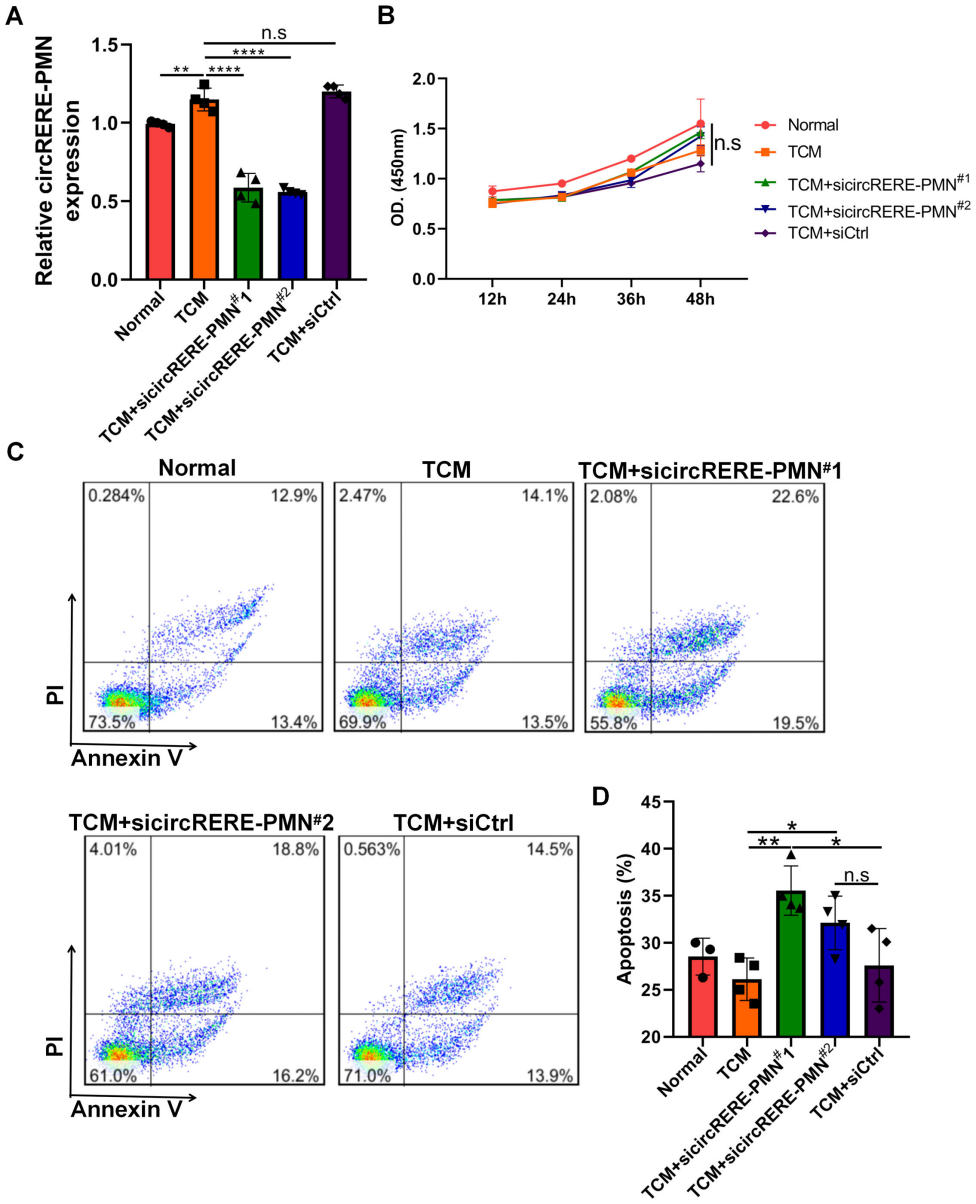
CircRERE-PMN participates in the apoptosis of MLE-12 cells. **(A)** RT-qPCR analysis for the circRERE-PMN expression level after circRERE-PMN knockdown. **(B)** CCK8 analysis for the proliferation of MLE-12 cells after circRERE-PMN knockdown. **(C)** Flow cytometry analysis of MLE-12 cells after circRERE-PMN knockdown. **(D)** Statistical analysis the percentages of apoptotic cells after circRERE-PMN knockdown. *p < 0.05, **p < 0.01, ****p < 0.0001, n.s, no significance.

Due to the significant difference in circRERE-PMN levels between normal and pre-metastatic lung tissues and its abundant presence in alveolar epithelial cells, further research was carried out to explore its role in these predominant lung cells. Initial observations showed that MLE-12 cells express circRERE-PMN to some extent, with a clear increase under B16F10-conditioned medium (TCM) (**Figure 6A**). Given the increased quantity of alveolar epithelial cells in pre-metastatic lungs and the potential influence of differentially expressed circRNAs on apoptosis pathways, we investigated the impact of circRERE-PMN on apoptosis and proliferation in MLE-12 cells. The results indicated that while MLE-12 cell proliferation was unaffected, apoptosis increased following the silencing of circRERE-PMN, as shown in **Figures 6B–D**. This suggests that circRERE-PMN does not significantly influence MLE-12 cell proliferation but does inhibit apoptosis.

### CircRERE-PMN is essential for regulating various chemokines and cytokines within alveolar epithelial cells

**Figure 7:**
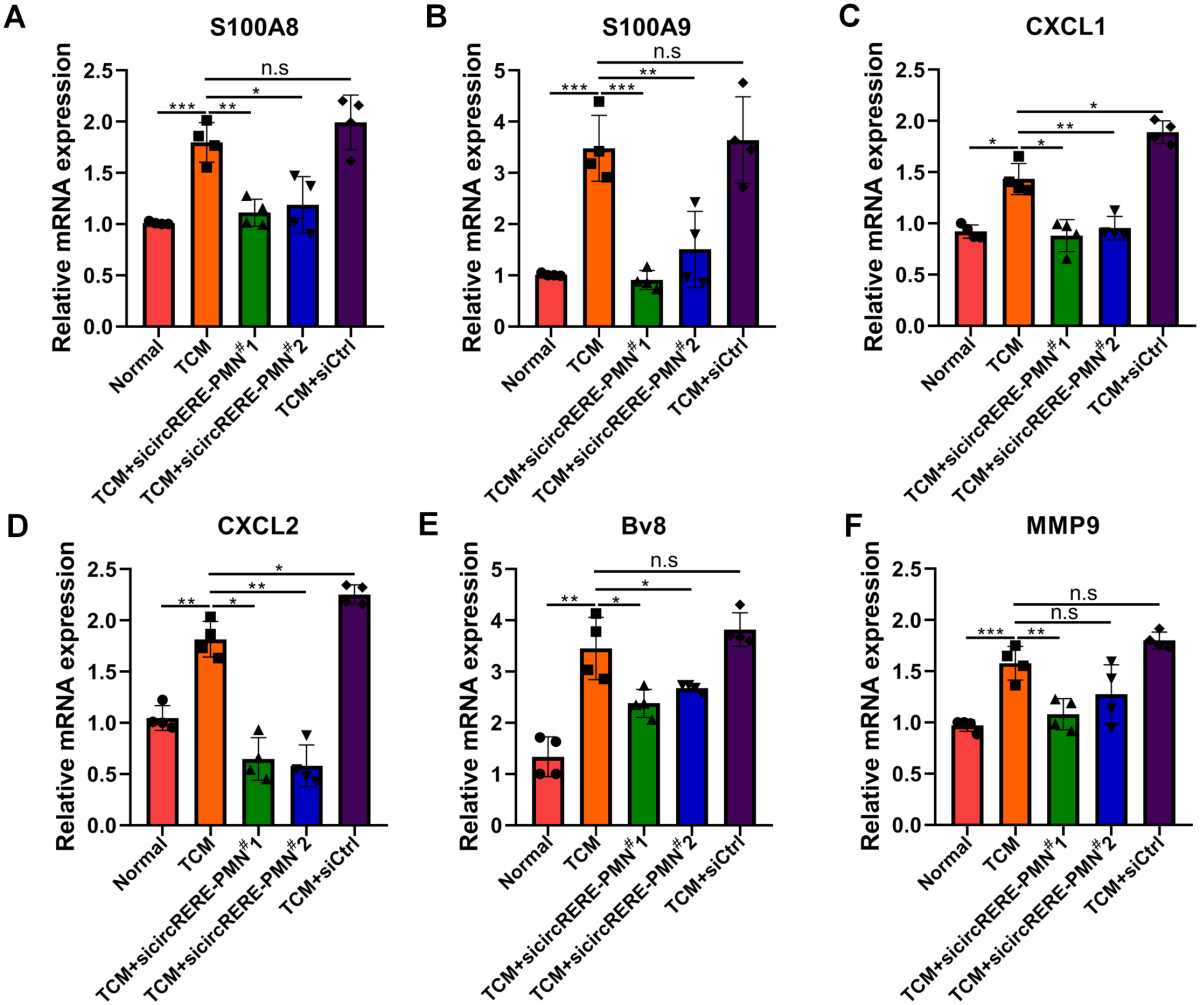
CircRERE-PMN influences the expression of a range of chemokines and cytokines within alveolar epithelial cells. **(A–F)** RT-qPCR analysis of the expression of S100A8 **(A)**, S100A9 **(B)**, CXCL1 **(C)**, CXCL2 **(D)**, Bv8 **(E)**, and MMP9 **(F)** after circRERE-PMN knockdown in MLE-12 cells. *p < 0.05, **p < 0.01, ***p < 0.001, n.s, no significance.

The connection between inflammation and the PMN, along with KEGG analysis indicating circRNA involvement in inflammation, prompted an examination of circRERE-PMN’s effect on the expression of inflammatory chemokines and cytokines. First, the role of circRERE-PMN in the expression of inflammation-related proteins S100A8/A9 in alveolar epithelial cells, which are closely related to the PMN, was investigated. It was observed that the suppression of circRERE-PMN significantly reduced S100A8/A9 levels in MLE-12 cells (**Figures 7A, B**). Additionally, alveolar epithelial cells were reported to contribute to establishing a pre-metastatic lung environment by attracting neutrophils through the secretion of cytokines, chemokines, and vesicular substances. Consequently, the chemokines CXCL1 and CXCL2, associated with neutrophil recruitment were detected, and they were significantly decreased in MLE-12 cells following circRERE-PMN silencing (**Figures 7C, D**). Moreover, cytokines involved in creating a pre-metastatic microenvironment, including Bv8 and MMP9, were also observed to decrease to some extent, as indicated in **Figures 7E, F**. These results collectively underscore the crucial role of circRERE-PMN in regulating multiple chemokines and cytokines vital for establishing a pre-metastatic microenvironment.

### Prediction of the interactions between circRERE-PMN and RNA-binding proteins

**Figure 8:**
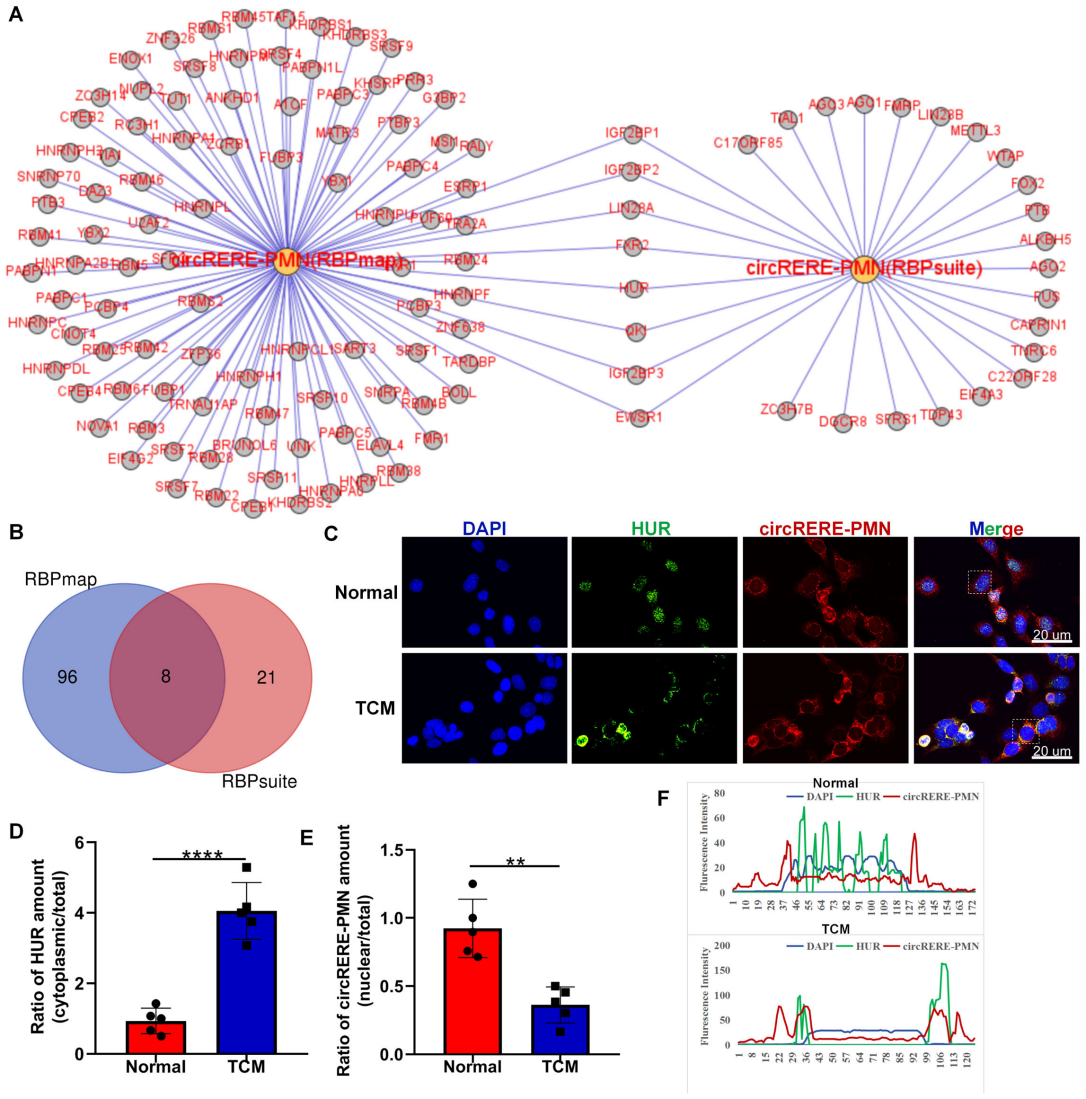
Interaction analysis between circRERE-PMN and its potential RBPs using RBPmap and RBPsuite. **(A)** Predicted RBPs targeted by circRERE-PMN were identified using RBPmap and RBPsuite. **(B)** A Venn diagram was utilized to delineate the common and unique RBPs targeted by circRERE-PMN. **(C)** FISH and immunofluorescence were used to measure circRERE-PMN (red) and HUR (green) in MLE-12 cells (Normal) and B16F10-conditioned medium treated MLE-12 cells (TCM). Scale bar, 20 μm. **(D)** The cytoplasmic distribution of HuR was quantified by densitometry analysis using Image J software. **(E)** The nuclear distribution of circRERE-PMN was quantified by densitometry analysis using Image J software. **(F)** The fluorescence intensity of HUR and circRERE-PMN from outlined cells in the merged images of panel **(C)**, analyzed by Image J software (blue line: DAPI; green line: HUR; red line: circRERE-PMN). **p < 0.01, ****p < 0.0001.

Given the recognized function of circRNAs in interacting with RBPs for essential activities (21), we predicted the potential RBPs that might bind to circRERE-PMN using RBPmap and RBPsuite, both of which are specialized in identifying RBPs that could interact with specific RNAs (22, 23). As these tools are based on human-derived data, our search was conducted using the human homologous sequence of circRERE-PMN, which is highly homologous. Our findings indicated that out of the 37 proteins identified by RBPsuite, 29 were predicted to interact with has-circRERE-PMN (**Figure 8A**). Furthermore, RBPmap predicted 104 RBPs to be associated with has-circRERE-PMN. Notably, 8 RBPs were consistently predicted by both databases, as illustrated in **Figures 8A, B**. Based on the RBP binding scores obtained from RBPmap and RBPsuite analyses, FXR2, QKI, and HUR demonstrated strong binding affinity towards circRERE-PMN (**Table 4**). Due to HUR’s documented pivotal roles in the regulation of inflammation and apoptosis, we examined the distribution of protein HUR and the interaction of HUR with circRERE-PMN in MLE-12 cells exposed to TCM. Interestingly, immunofluorescence microscopy revealed nuclear compartmentalization of HUR in normal cells, whereas in TCM-exposed cells, HUR preferred to translocate to the cytoplasm (**Figures 8C, D**). Moreover, results showed that nuclear circRERE-PMN signals were overt in normal MLE-12 cells compared with that in the TCM-treated MLE-12 cells (**Figures 8C, E**), which both exhibited a significant co-localization with HUR (**Figures 8C, F**). These findings suggest a potential interaction between circRERE-PMN and the downstream target HUR.

**Table 4:** Max binding scores of RBPs to circRERE-PMN.

RBPs	Max binding score	
	RBPsuite	RBPmap
FXR2	0.8813393	3.571
QKI	0.7586525	1.913
HUR	0.74990827	2.769
LIN28A	0.7166275	3.311
IGF2BP3	0.71051013	3.463
EWSR1	0.69480145	2.22
IGF2BP2	0.6936594	3.425
IGF2BP1	0.6514302	1.761

## Discussion

The formation of a pre-metastatic microenvironment is essential for cancer dissemination, as it significantly impacts the capture, survival, and proliferation of circulating tumor cells at future metastatic sites, thus facilitating distant metastasis. Numerous non-coding RNAs, including circRNAs, have been linked to the PMN. Given their conservation, stability, abundance, and specific expression patterns, circRNAs are posited to play crucial roles in developing PMN. Our study is the first to profile circRNA expression in pre-metastatic lungs. Validation of the top 6 altered circRNAs was accomplished using RT-qPCR, and functional predictions were made through GO and KEGG pathway analyses. Our biological investigation revealed that the increased presence of circRERE-PMN in pre-metastatic lungs suppressed apoptosis in alveolar epithelial cells and elevated the production of various cytokines and chemokines, underscoring the significant role of circRNAs in lung preconditioning for metastasis and their potential as biomarkers for the early detection of lung metastases in cancer.

CircRNAs, primarily thought to act as microRNA sponges or splicing regulators, are increasingly acknowledged for their influence on the gene expression of their host genes at various stages, including transcription, post-transcription, translation, and post-translation. As a result, circRNAs often exhibit either synergistic or antagonistic functions concerning their host genes. Through functional studies, we have demonstrated that circRERE-PMN is involved in the programmed cell death of alveolar epithelial cells. This specific circRNA is derived from the arginine-glutamic acid dipeptide repeats gene (RERE) via back-splicing of the second exon of RERE, though its precise functional mechanisms remain unexplored. RERE is a nuclear receptor coregulator involved in gene regulation through transcriptional control or interaction with HDAC1/2 (24, 25). In humans, RERE is associated with neurological development disorders, sometimes accompanied by structural abnormalities in the eyes, brain, kidneys, and heart (26). A deficiency in RERE can lead to orofacial clefts, and in mice, the absence of RERE results in retinal degeneration and optic nerve atrophy (27). Mechanistic studies have shown that RERE is crucial for retinal development, and its absence triggers apoptosis in retinal cells without significantly affecting the proliferation of these cells’ progenitors. Another circRERE, formed by back-splicing of exons 5-8, is found to be reduced in osteoarthritis cartilage and is associated with increased apoptosis and abnormal β-catenin ubiquitination and degradation via the miR-195-5p/IRF2BPL axis (28). The circRERE (has_circ_12952), produced from exon 3 of the RERE gene, has been shown to enhance the type I interferon signaling pathway and cytokine production, supporting anti-tumor immunity in colorectal cancer (29). As previously mentioned, circRNAs can either mirror or counteract the functions of their host genes. Our research also indicates that circRERE-PMN is involved in cell apoptosis and cytokine production. Due to their greater stability and resistance to exonuclease degradation compared to linear RNAs, circRNAs are emerging as promising candidates for diagnostic biomarkers and therapeutic interventions. For example, hsa_circ_0000096 modulated VEGF expression and emerged as a prospective biomarker for gastric carcinoma (30); the expression of circ-IARS in pancreatic cancer exhibited a positive association with tumor size, metastasis, and TNM staging, while demonstrating a negative correlation with prognosis (15). All in all, multiple studies have identified unique circRNA expression profiles linked to specific cancer types and patient outcomes, and a significant amount of pre-clinical research has driven clinical trials focusing on circRNAs as potential therapeutics (31). Our findings suggest a significant increase in circRERE-PMN levels in lungs before metastasis, highlighting the need to evaluate its clinical predictive value for lung metastasis monitoring and its potential as a therapeutic target. Additionally, the mechanisms by which circRERE-PMN operates in pre-metastatic lungs require further investigation.

RBPs are key trans factors associated with specific cis elements present in mRNA. Binding to RBPs is considered a significant pathway for circRNA function, including the regulation of the genesis, translation, and transcription of target genes. We observed that circRERE-PMN could bind with downstream target HUR. HUR is a ubiquitous protein that mainly determines the fate of cytoplasmic mRNA. Aberrant expression of HUR may lead to diseases such as cancer, neurodegenerative disorders, and inflammatory diseases (32). By interacting with targeted mRNAs, HUR is responsible for cellular apoptosis, differentiation, senescence, proliferation, and responses to inflammatory and immune stimuli. In cancers, HUR is broadly elevated in virtually all tested malignancies and plays a causal role in tumor development and progression (33). For instance, overexpression of HUR resulted in an inflammatory response and facilitated tumor development in pancreatic tumorigenesis (34). HUR knockout in tumor-associated macrophages (TAMs) altered the migration, chemoattraction, and chemokine/cytokine profiles of malignant glioma in mice (35). Inhibiting HUR significantly upregulated the expression of TRAF1, IKK-α, NIK, p52, pro-Caspase 3, and PARP in lymphoblastoid cell lines and B lymphocytes, thus increasing the inflammatory response and apoptosis (36).

In summary, our research offers an initial insight into the expression patterns of circRNAs in pre-metastatic lungs. Importantly, we are the first to identify the role of circRERE-PMN in modulating cell apoptosis, as well as cytokine and chemokine production in alveolar epithelial cells, which are closely linked to pre-metastatic conditions. Essentially, our results highlight the critical role of circRNAs in pre-metastatic lungs and propose that targeting circRERE-PMN might provide a novel approach for diagnosing or treating lung metastasis.

## Data Availability

The data presented in the study are deposited in the GEO repository, accession number GSE274656.
